# Supplementary material for: Competing-Risk Analysis of Death and End Stage Kidney Disease by Hyperkalaemia Status in Non-Dialysis Chronic Kidney Disease Patients Receiving Stable Nephrology Care
Source: J Clin Med. 2018 Dec 1;7(12):499. doi: 10.3390/jcm7120499 (PMC6306758; doi:10.3390/jcm7120499)
Supplement: Supplementary file 1 [file jcm-07-00499-s001.pdf]

Competing-Risk Analysis of Death and ESKD by Hyperkalemia Status in Non-Dialysis CKD Patients Receiving Stable Nephrology Care.

Provenzano M et al.

APPENDIX

Investigators in each cohort study……..pg 2

Description of pooled cohorts………….pg 3

Table S1……………………………….pg 5

Figure S1……………………………….pg 6

Table S2……………………………….pg 7

Table S3……………………………….pg 8

COMPLETE LIST OF INVESTIGATORS IN EACH COHORT STUDY

TArget BP LEvels in CKD (TABLE-CKD) Study Group:
L. De Nicola, R. Minutolo, P. Zamboli, F., C. Iodice, S. Borrelli, P. Chiodini, S. Signoriello, C. Gallo, G. Conte (Napoli-2° University Medical School); T. Materiale, B. Minale, C. Paglionico (Napoli-ASL NA 1); B. Cianciaruso, A. Pota (Napoli-University Medical School Federico II); F. Nappi, F. Avella (Nola-Hospital); B.R. Di Iorio, V. Bellizzi (Solofra-Hospital); R. Cestaro (Sapri-Hospital); V. Martignetti, L. Morrone (Benevento-Hospital); A. Lupo, C. Abaterusso (Verona-University Medical School); C. Donadio (Pisa-University Medical School); M. Bonomini, V. Sirolli (Chieti-University Medical School); F. Casino, T. Lopez (Matera-Hospital); F. Detomaso, M. Giannattasio (Putignano-Hospital); M. Virgilio, G. Tarantino (Molfetta-Hospital); C. Cristofano, S. Tuccillo, S. Chimienti (Manduria-Hospital); F. Petrarulo, V. Giancaspro (Bari-Di Venere Hospital); M. Strippoli (Bari-University Medical School); E. Laraia (Bari-S. Rita Hospital); M. Gallucci, B. Gigante (Galatina-Hospital); C. Lodeserto, D. Santese (Taranto-Hospital); A. Montanaro, R. Giordano (Martina Franca-Hospital); A. Caglioti, G. Fuiano (Catanzaro-University Medical School); C. Zoccali, G. Caridi, M. Postorino (Reggio Calabria-CNR); V. Savica, P. Monardo (Messina-Hospital); G. Bellinghieri, D. Santoro (Messina-University Medical School); and P. Castellino, F. Rapisarda, P. Fatuzzo, A. Messina, (Catania-University Medical School).

REporting COmorbidities in non-dialysis Renal Disease population in ITaly (RECORD-IT) Study Group:
A. Dal Canton, V. Esposito (University of Pavia. Pavia), M. Formica (S. Croce e Carle Hospital, Ceva), G. Segoloni (University of Torino, Torino), M. Gallieni (S. Carlo Borromeo Hospital, Milano), F. Locatelli (A. Manzoni Hospital, Lecco), R. Tarchini (C. Poma Hospital, Cremona), G. Meneghel (Dolo General Hospital, Dolo), L. Oldrizzi (Fracastoro Hospital, San Bonifacio), M. Cossu (SS Annunziata Hospital, Sassari), S. Di Giulio (S. Camillo Forlanini Hospital, Roma), M. Malaguti (S. Paolo Hospital, Civitavecchia), F. Pizzarelli (S. M. Annunziata, Firenze), G. Quintaliani (S. Maria della Misericordia Hospital, Perugia), B. Cianciaruso, A. Pisani (University “Federico II”, Naples), G. Conte, L. De Nicola, R. Minutolo (Second University of Naples, Naples), R. Bonofiglio (Ospedale dell’Annunziata, Cosenza), G. Fuiano (University of Catanzaro, Catanzaro), G. Grandaliano (University of Foggia, Foggia), G. Bellinghieri, D. Santoro (University of Messina, Messina).

NEPHROlogy clinic at FEDERICO II University (NEPHRO-FEDERICO II)
B. Cianciaruso, A. Pota, L. DI Micco, M. Sabbatini, A. Pisani, D. Di Giuseppe (University “Federico II”, Naples).

NEPHROlogy clinic of SUN (NEPHRO- SUN)

L. De Nicola, M. Provenzano, P. Chiodini, S. Borrelli, C. Garofalo, M. Pacilio, M.E. Liberti, A. Sagliocca, G. Conte, R. Minutolo (Napoli-Second University-Med School)

DESCRIPTION OF THE FOUR POOLED COHORTS

The single four cohort studies (Target Blood pressure LEvel in CKD, **TABLE-CKD** study[1]; Nephrology study at Second University of Naples-**NEPHRO SUN** study[2]; REport of COmorbidities in non-Dialysis Renal Disease Population in Italy, **RECORD-IT** study[3]; Nephrology study at University Federico II-**NEPHRO-FEDERICO II** study[4]) had been originally approved by Institutional Review Boards and patients gave written consent to use their clinical data. Cohorts were originally built to collect prospective information of patients referred to CKD clinics.

The four studies shared same procedures, endpoints (ESKD and all-cause death), as well as inclusion (ND-CKD patients referred to nephrology clinic) and exclusion criteria (renal replacement therapy, AKI, active malignancy, life expectancy <6 months, advanced liver or heart disease).

In the four studies, nephrologists collected medical history including CKD cause, cardiovascular disease: stroke, coronary heart disease, heart failure, peripheral vascular disease. They also performed physical examination, and registered laboratory results, blood pressure, therapy and events in anonymous electronic case reports that were periodically sent to the coordinating center for quality analysis and storage.

Laboratory protocols were standardized with in-house analyses. Proteinuria was quantified by 24h-urine collections; collection was considered inaccurate, and repeated, if creatinine excretion was outside the expected range[5]. Estimated GFR (eGFR) was calculated by the CKD-EPI equation with serum creatinine levels reduced by 5% according to Skali et al. because creatinine was not standardized to isotope-dilution mass spectrometry values[6].

REFERENCES

1. De Nicola L, Chiodini P, Zoccali C, et al. SIN-TABLE CKD Study Group: Prognosis of CKD patients receiving outpatient nephrology care in Italy. Clin J Am Soc Nephrol 2011, 6:2421-2428.
2. De Nicola L, Provenzano M, Chiodini P, et al. Independent Role of Underlying Kidney Disease on Renal Prognosis of Patients with Chronic Kidney Disease under Nephrology Care. *PLoS One* 2015, 10:e0127071.
3. Minutolo R, Locatelli F, Gallieni M, et al. REport of COmorbidities in non-Dialysis Renal Disease Population in Italy (RECORD-IT) Study Group. Anaemia management in non-dialysis chronic kidney disease (CKD) patients: a multicenter prospective study in renal clinics. *Nephrol Dial Transplant* 2013, 28:3035-3045
4. Cianciaruso B, Pota A, Bellizzi V, et al. Effect of a Low-Versus Moderate-Protein Diet on Progression of CKD: Follow-up of a Randomized Controlled Trial. *Am J Kidney Dis* 2009, 54:1052-1061
5. Dwyer J, Kenler SR. Assessment of nutritional status in renal disease. In: Mitch WE, Klahr S (eds). *Nutrition and the Kidney*, 2nd edn Boston, MA, Little, Brown and Company, pg 61-95, 1993
6. Skali H, Uno H, Levey AS, et al. Prognostic assessment of estimated glomerular filtration rate by the new chronic kidney disease epidemiology collaboration equation in comparison with the modification of diet in renal disease study equation. *Am Heart J* 2011, 162:548-554

.

Table S1. Basal features and outcome of patients excluded from the study due to missed visit 2 in comparison with included patients.

	**Excluded**	**Included**	**P**
N	370	2443	-
Age (years)	69.5±14.6	65.1±14.6	<0.001
Males (%)	56.0	58.3	0.395
BMI (kg/m^2)	26.5±4.2	27.8±5.1	<0.001
Diabetes (%)	35.1	27.9	0.004
CV disease (%)	38.1	35.9	0.401
Smoking (%)	8.6	13.2	0.015
Systolic BP (mmHg)	140±18	139±20	0.277
Diastolic BP (mmHg)	81±10	79±11	0.051
Potassium (mEq/L)	4.81±0.68	4.81±0.62	0.960
Phosphate (mg/dL)	4.1±1.0	3.8±0.8	<0.001
Hemoglobin (g/dL)	12.0±1.9	12.7±1.8	<0.001
eGFR (mL/min/1.73m^2)	24.3±14.3	35.0±17.3	<0.001
Proteinuria (g/24h)	0.83 [0.30-1.68]	0.40 [0.14-1.21]	<0.001
RASI use	67.1	70.6	0.178
Outcome			
First-year rate ESKD	26.8 (21.2-33.4)	7.5 (6.4-8.7)	<0.001
First-year rate Death	14.6 (10.5- 19.6)	3.5 (2.8-4.4)	<0.001

BMI, body mass index; BP, blood pressure; CV, cardiovascular; eGFR, estimated glomerular filtration rate by EPI equation; RASI, renin-angiotensin system inhibitors. Continuous variables are reported as mean±SD or median and [IQR] according to their distribution.

Figure S1. Changes of hyperkalemia status (serum K ≥5.0 mEq/L) over the two study visits with dependent categorization of the four subgroups of patient.

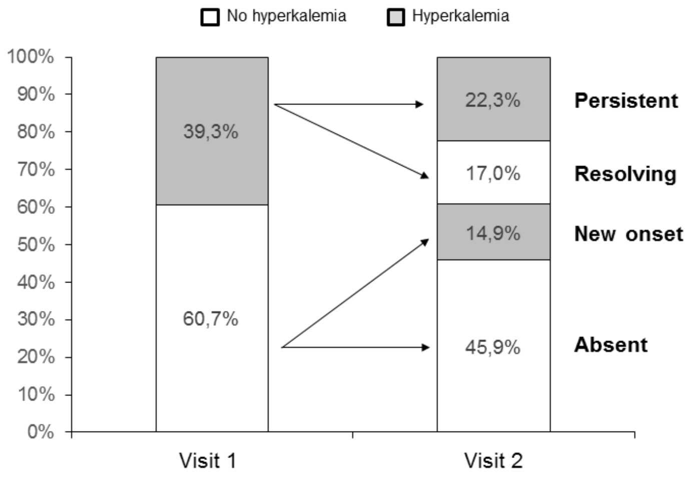

Table S2. Full models on risk of ESKD and all-cause death.

	ESKD			Death		
	**sHR**	**95% CI**	**P**	**sHR**	**95% CI**	**P**
Age (year)	**0.96**	**0.96-0.97**	**<0.001**	**1.09**	**1.07-1.10**	**<0.001**
Male gender	**1.61**	**1.32-1.96**	**<0.001**	1.10	0.86-1.41	0.431
BMI (kg/m^2)	1.00	0.98-1.02	0.980	1.01	0.98-1.03	0.770
Diabetes	0.79	0.62-1.04	0.063	**1.38**	**1.08-1.77**	**0.010**
CV Disease	**1.28**	**1.04-1.58**	**0.019**	1.08	0.85-1.36	0.540
Systolic BP (mmHg)	**1.01**	**1.005-1.015**	**<0.001**	0.99	0.98-1.01	0.240
Hyperkalemia						
Absent	Ref.	-	-	Ref.	-	-
Resolving	0.98	0.72-1.33	0.890	1.01	0.73-1.36	0.901
New onset	**1.34**	**1.05-1.72**	**0.020**	0.94	0.64-1.38	0.760
Persistent	**1.27**	**1.02-1.58**	**0.036**	0.91	0.67-1.25	0.560
Hemoglobin (g/dL)	**0.89**	**0.83-0.95**	**0.001**	0.98	0.90-1.06	0.580
eGFR (ml/min/1.73m^2)	**0.91**	**0.90-0.92**	**<0.001**	0.99	0.98-1.01	0.784
eGFR change (mL/min)	1.01	0.99-1.02	0.550	0.99	0.98-1.01	0.323
Proteinuria (g/24h)	**1.19**	**1.09-1.29**	**<0.001**	1.03	0.98-1.09	0.220
Use of RASI						
Both visits	Ref.	-	-	Ref.	-	-
Only visit 2	1.06	0.74-1.53	0.740	1.15	0.75-1.76	0.522
Only visit 1	1.08	0.81-1.44	0.600	**2.02**	**1.37-2.99**	**<0.001**
None visit	**1.30**	**1.03-1.65**	**0.030**	1.30	0.97-1.73	0.089

sHR, sub-hazard ratio; CI, confidence interval; BMI, body mass index; BP, blood pressure; CV, cardiovascular; eGFR, estimated glomerular filtration rate by EPI equation; RASI, renin-angiotensin system inhibitors. Models are stratified by cohort. Bold indicates statistical significance.

Table S3. Determinants of ESKD and all cause death over the first year of follow up in whole original population (N=2813).

	**ESKD**		**All-cause death**	
	**sHR**	**(95%-CI)**	**sHR**	**(95%-CI)**
Age (years)	**0.97**	**0.96-0.97**	**1.09**	**1.08-1.10**
Male gender	**1.46**	**1.23-1.74**	1.10	0.90-1.35
BMI (kg/m^2)	0.99	0.97-1.01	0.99	0.98-1.02
Diabetes	0.87	0.71-1.06	**1.33**	**1.08-1.63**
CV disease	1.17	0.98-1.40	1.18	0.97-1.44
Systolic BP (mmHg)	**1.006**	**1.002-1.010**	1.00	0.99-1.01
eGFR ($mL/min/1.73m^2$)	**0.91**	**0.90-0.92**	1.01	0.99-1.01
Proteinuria (g/24h)	**1.20**	**1.12-1.30**	**1.06**	**1.01-1.10**
RASI use	0.89	0.74-1.07	0.86	0.70-1.06
Haemoglobin, g/dL	**0.93**	**0.88-0.98**	0.94	0.88-1.01
Potassium (mEq/L)	0.99	0.87-1.12	0.90	0.76-1.07

The variables included in the models are from Visit1.
Models are stratified by cohort. Bold indicates statistical significance.
sHR, sub-hazard ratio; CI, confidence interval; BMI, body mass index; BP, blood pressure; CV, cardiovascular; eGFR, estimated glomerular filtration rate by EPI equation; RASI, renin-angiotensin system inhibitors.
